# Hyperammonemia reduces the populations of beneficial lactobacilli and bifidobacteria, disrupting the metabolic balance of the gut microbiome in rats

**DOI:** 10.3389/fmicb.2026.1771709

**Published:** 2026-03-27

**Authors:** Lola Giner-Pérez, Vicente Felipo, Paula Izquierdo-Altarejos, Marta Llansola, Gaspar Pérez Martínez

**Affiliations:** 1 Laboratory of Lactic Acid Bacteria and Probiotics, Instituto de Agroquímica y Tecnología de Alimentos (IATA-CSIC), Valencia, Spain; 2 Laboratory of Neurobiology, Centro de Investigación Príncipe Felipe, (CIPF), Valencia, Spain; 3 INCLIVA Instituto de Investigación Sanitaria, Valencia, Spain

**Keywords:** *Alistipes*, gut microbiota, hyperammonemia, lactic acid bacteria and bifidobacteria, liver disease, metabolic modules, SCFA (short chain fatty acids)

## Abstract

**Introduction:**

The gut microbiome (GM) plays a critical role in metabolic and neurological health and is implicated in hepatic encephalopathy (HE). Chronic hyperammonemia (HA), a major contributor to cognitive and motor impairment in HE, may influence GM structure and function, yet its specific efects in GM remain unclear.

**Methods:**

Here, it was investigated how chronic HA alters the GM using a rat model fed an ammonia-enriched diet for 4 weeks. Fecal microbiota profiles obtained by 16S rRNA gene sequencing revealed marked taxonomic shifts in HA rats, with beta-diversity showing clear separation from controls.

**Results:**

Genera within the Lachnospiraceae family and *Alistipes* genus were enriched in HA rats, while lactic acid-producing and xylanolytic Firmicutes were reduced. Network analysis identified *Alistipes* as a central node in the HA microbiome. Predicted metabolic functions were significantly altered, showing negative associations between HA and pathways related to the pyruvate dehydrogenase complex, sucrose and urea degradation, and 4-aminobutyrate (GABA) degradation. Consistent with these predictions, fecal short-chain fatty acid (SCFA) analysis revealed reduced acetic and butyric acid, alongside increased valeric and isobutyric acid levels. The predicted GABA levels increasement by GM would activate GABA receptors in immune cells and would also contribute to peripheral inflammation and, eventually, neuroinflammation.

**Conclusions:**

Together, these findings demonstrate that chronic HA reshapes GM composition, disrupts key metabolic pathways, and alters SCFA profiles, providing mechanistic insight into how HA- associated dysbiosis may contribute to the metabolic, immune, and neurological dysfunction characteristic of HE.

## 1 Introduction

Hepatic encephalopathy (HE) is a neuropsychiatric syndrome affecting cerebral function due to pre-existing liver dysfunction, ranging from minimal to severe in patients with chronic liver disease (Felipo, 2013). Treating HE patients with rifaximin, a non-systemic antibiotic that targets the gut, restores cognitive function and reduces the risk of recurrent HE. This suggests that the gut microbiome (GM) plays a significant role in the cognitive aspects of this disease (Bajaj, 2014; Mangas-Losada et al., 2019). In order to develop effective treatments for cognitive and motor impairment in HE patients, it is necessary to understand the mechanisms that lead to HE, including GM changes. Chronic hyperammonemia (HA) is a major contributor to cognitive and motor impairment in cirrhotic patients with HE (Weissenborn et al., 2005), as demonstrated in animal models simulating human HA (Felipo et al., 1988; Jover et al., 2006). In cirrhotic patients, HA is induced by impaired ammonia detoxification through the urea cycle in the injured liver. Ammonia is produced by amino acid degradation in the small intestine and by bacteria in the colon, which degrade amino acids, as well as by some bacteria species that produce urease. Cirrhotic patients are therefore likely to have increased levels of ammonia in the gut, in which increased glutaminase activity contributes to the increase in ammonia levels, both in the gut and in systemic circulation (Damink et al., 2009; Romero-Gómez et al., 2009). Neuroinflammation is mainly induced by transfer of peripheral inflammation induced by HA to the brain. In rat models, HA produces neuroinflammation that alters the glutamatergic neurotransmission in the hippocampus and GABAergic neurotransmission in the cerebellum, leading to cognitive impairment and motor alterations (Balzano et al., 2020). Although peripheral inflammation in cirrhosis is primarily driven by liver injury, HA further amplifies both peripheral and central inflammatory responses (Balzano et al., 2020). Consequently, changes in GM induced by HA could contribute to peripheral inflammation and brain dysfunction, which is supported by the fact that rifaximin improved both, peripheral inflammation and neurological function in HE patients (Mangas-Losada et al., 2019).

A previous study investigated changes in the microbiome associated with chronic HA in mice (Abdelmohcine et al., 2023) and reported a decreased Shannon index, as well as an increased abundance of *Enterobacteriaceae, Streptococcaceae*, and *Enterococcaceae* whereas *Lactobacillus* abundance is decreased. Moreover, it has also been demonstrated that *Lactobacillus helveticus* NS8 improves cognitive decline and anxiety-like behavior in HA rats (Luo et al., 2014). We hypothesized that the increased intestinal ammonia could contribute to alterations in the GM and in its metabolic functions, which in turn might explain the occurrence of peripheral and neuroinflammation. To this end, this study examined the impact of chronic HA on the GM in rats and analyzed the taxonomic and the likely metabolic changes induced by HA.

## 2 Materials and methods

### 2.1 Experimental design

Male Wistar rats (120-140 g, Charles River Laboratories, Barcelona, Spain) were made hyperammonemic by feeding them a diet containing standard diet supplemented with 30% ammonium acetate (Felipo et al., 1988; Taoro-Gonzalez et al., 2018). The fecal samples were obtained after 4-5 weeks, when rats present chronic hyperammonemia. The experiments were approved by the Comité de Etica y Experimentación Animal (CEEA) of the CIPF (approval number 2024-24) and by Conselleria de Agricultura, Generalitat Valenciana (approval code 2024/VSC/PEA/0268), and performed according to the Directive of the European Commission (2010/63/EU) for the care and management of experimental animals and complied with the ARRIVE guidelines for animal research.

Two groups were established: healthy rats (*n* = 14) and HA rats (*n* = 13). The experiment was run in three batches with 8 rats in batch B1, 7 in batch B2 and 12 rats in batch B3.

### 2.2 DNA extraction and library preparation

Fecal samples were flash-frozen in liquid nitrogen and preserved at −80 °C. DNA was extracted using a magnetic bead-assisted technique following the manufacturer's protocol (Maxwell RSC Instrument using Maxwell RSC Pure Food GMO and authentication kit; Promega, Spain). DNA concentration was quantified using a Qubit 2.0 fluorometer (Life Technology, Carlsbad, CA, USA) and adjusted to 5 ng/μL. Negative controls were included. Sequencing of the V3-V4 hypervariable regions of the 16S gene was performed as in Giner-Pérez et al. (2025).

### 2.3 Sequencing processing and bacterial profiling

Pre-processing was performed using the DADA2 (v1.18.0) pipeline to infer amplicon sequence variants (ASVs) from the SILVA database (v138.1). Primer sequences were trimmed, forward and reverse reads were truncated to 280 and 265 bases. Fragments between 400-427 bp were selected *in silico*. All other parameters were set to default.

Filtering and statistical analyses were carried out using R programming language (v4.1.2) in RStudio (v2021.09.0). The decontam package (v1.14.0) was employed to identify potential contaminants. ASVs confirmed to be contaminants searching in existing literature were removed. Low prevalent ASVs were excluded. ASV counts were transformed to relative abundances for differential abundance analysis, and to central log-ratio (clr) for further statistical analyses. One sample with few counts was excluded from the analysis. Remaining samples reached saturation and sequencing depth difference was below one order of magnitude (27438–49421).

### 2.4 Alpha and beta diversity

Alpha diversity indices (Shannon, Chao1, and Fisher) were calculated from raw sample counts. Statistical differences between groups and batch effects were assessed using linear mixed-effects modeling. *P*-values were adjusted using the Benjamini-Hochberg method. Plots were generated with ggplot2 (v3.4.2). Data were clr-transformed and batch effects removed using removeBatchEffect from the limma package (v3.50.3). For beta diversity, Euclidean distances were computed with vegdist (v2.6.4) and visualized via PCoA. PERMANOVA was performed with the adonis function (v2.6.4) using 999 permutations and batch stratification.

### 2.5 Predicted functional annotation

Predicted the functionality of the GM was obtained using PICRUSt2 algorithm (https://github.com/picrust/picrust2) based on ASV counts. Omixer-rpmR classified predicted KO counts to gut metabolic modules (GMMs), using the coverage cut-off by default (Douglas et al., 2020). To trace tryptophan (trp) metabolism, modules were manually added to the Omixer-rpmR clustered databases as in Giner-Pérez et al. (2025).

### 2.6 Correlation and Network analyses

MetadeconfoundR (Till Birkner and Sofia Kirke Forslund-Startceva, 2021) (v0.2.8) was used for assessing associations with ASVs and GMMs across metadata and detection of confounded covariates. Batch was included as a random variable.

Networks were created using SparCC correlation-based networks with the netConstruct function (NetCoMi, v1.1.0). Parameters included taxonomic rank (genus), zero method (clr), and sparsity method (t-test). The resulting networks were analyzed using the netAnalyze function (NetCoMi) with the cluster_fast_greedy method (Peschel et al., 2021). Statistical significance of correlations and network properties was assessed. A fixed seed value of 1,23,456 ensured reproducibility. A permutation-based null model (*n* = 500 label permutations) was implemented by randomly shuffling group labels prior to network reconstruction. Density differences between permuted groups were used to generate a null distribution.

### 2.7 Quantification of fecal short chain fatty acids (SCFA) and neurotransmitters (GABA and glutamate)

The concentration of SCFA and neurotransmitter amino acids in feces was measured by LCMS with an EXION (Shimatzu) HPLC system coupled to a mass spectrometry detection system consisting of a QTRAP 4,500 triple quadrupole (AB Sciex, Ontario, Canada) equipped with electrospray ionization (ESI) ion source, controlled by the Analyst software, version 1.6.3. Feces (100 mg approximately) were homogenized during 2.5 min in 20 V (1:20 p/v) of H2O LC-MS grade using a homogenizer (Politron PT 1200E, from KINEMATIC AC, Lucerne, Switzerland). Then samples were centrifuged at 13.200 rpm for 10 min at 4°C and the supernatant was stored at −20° until use. The sample preparation proceeded as follows for SCFAs analysis: 30 μL of feces supernatants were derivatized with 10 μL of 1 M O-benzylhydroxylamine (O-BHA) (SIGMA) and 10 μL of 1 M N-(3-Dimethylaminopropyl)-N-ethylcarbodiimide-HCl (EDC) (SIGMA) prepared in freshly prepared pyridine buffer (270 μL of 12.1 M HCl þ 430 μL of pyridine in H_2_O to 5 mL, pH 5), in a fume hood. This derivatization mixture was agitated at 300 rpm for 10 min. Then 50 was added for extraction by agitation at 300 rpm for 30 min. Then, 100 μL of the organic phase was separated and evaporated at room temperature in a fume hood. The samples were reconstituted with 85 μL of 0.1% formic acid in H_2_O and 40 μL and injected in the HPLC under the following conditions: a Kinetex C18 100^*^4.6 mm 2.6 column from Phenomenex, at 40°C, was used. The mobile phase consisted of a two-phase gradient: 0.1% formic acid in water (A) and 0.1% formic acid in acetonitrile (B), as follows: 5-20% B 0-1 min, 20-50% B 1-5.5 min, 60% B 5.5-5.7 min, 80% B 6 min, 80% B 6.5 min, 5% B 6.6 min, 5% B 12 min, with a flow rate of 0.4 mL/min. The conditions of the mass spectrometer were: positive ionization mode, entrance potential 10, curtain gas 30, declustering potential 60 V, collision energy 15 eV, GAS1 40 and GAS2 50, 500°C and 4500 V in multiple reaction monitoring (MRM) mode with the following transitions for the quantification of the different SCFA: acetic acid 166.1 m/z > 91 m/z; propionic acid 180.1 m/z > 91 m/z; butyric acid and isobutyric acid 194.1 m/z > 91 m/z; valeric acid 208 m/z > 91 m/z and caproic acid 222 m/z > 91 m/z. A SCFA mixture standard curve, from 1 to 2,500 ng/mL (10-25,000 ng/mL of caproic acid), was prepared in H_2_O and derivatized and extracted as the samples to calculate SCFA concentrations.

For neurotransmitter amino acid analysis 100 μL of the fecal supernatant was used. Protein precipitation was performed by adding of 20 μL of trifluoroacetic acid and centrifuging at 4 °C during 10 min at 17,500 rcf. Then, 50 μL of this supernatant was injected into the same LC-MS system, using a Luna Omega Polar C18 (OOD- 4760-AN) 100^*^2.1mm3 μm (100 A) column from Phenomenex, at 30 °C. The mobile phase consisted of a two-phase gradient: 0.1% formic acid in water (A) and 0.1% formic acid in acetonitrile (B), as follows: 20% B 0-0.5 min, 20-80% B 0.5-5.0 min, 80% B 5.0-6.0 min, 80-20% B 6.0-6.1 min, 20% B 6.1-8 min, with a flow rate of 0.4 mL/min. ESI ion source in positive ionization mode was used with curtain gas 30, GAS1 40 and GAS2 60, 500°C and 4,500 V in multiple reaction monitoring (MRM) mode with the following conditions for each metabolite: (1) GABA, 104 m/z > 87, RT 0.6 EP 10, CE 15, DP 46, CXP 4; (2) Glutamate, 148 m/z > 84 m/z, RT 0.6 EP 10 CE 21 DP 41 CXP 6.

### 2.8 Statistical tests for significance of SCFA

Data were tested for normality with the Kolmogorov-Smirnov test and all data pass this normality test. Equality of variances was confirmed for all data. Then, unpaired *t*-test was used for comparison of control and HA groups. A confidence level of 95% was considered as significant.

## 3 Results

### 3.1 Control and HA rats have a different microbiome composition

**Figure 1:**
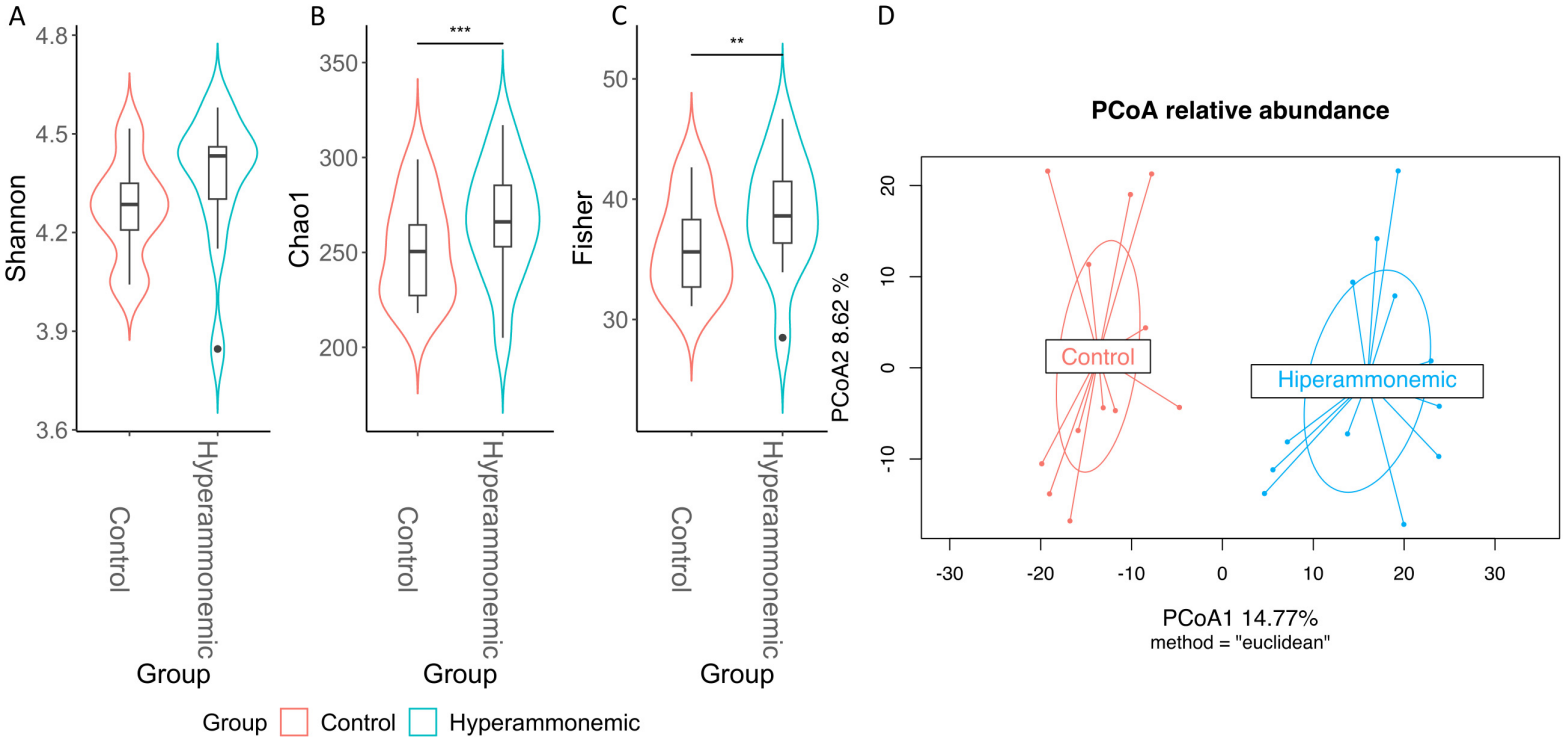
Alpha and beta diversity plots. Box plots illustrate alpha diversity indexes Shannon **(A)** Chaotropic 1 (Chao1) **(B)** and Fisher **(C)** in microbiomes of fecal samples from control and hyperamomonemic rats. Adjusted *p*-values of likelihood ratio test of nested models: <0.001 = ***, <0.01 = **, <0.05 = *. Principal Coordinates Analysis (PCoA) of Euclidean distances were used to compare the taxonomic diversity. The axis labels show the proportion of variance explained by each principal coordinate axis (%).

Alpha diversity is a measure of microbial community diversity, evenness and richness. The Shannon index (Figure 1A) revealed no significant differences between the groups (likelihood ratio test of nested models, Chi2 = 1.31, *p* = 0.73). However, significant differences between groups were observed when calculating Fisher's alpha index (Figure 1B) and Chao1 (Figure 1C) (Likelihood Ratio Test of Nested Models, Fisher: Chi2 = 26.964, *p* < 0.001; Chao1: Chi2 = 37.343, *p* < 0.001). Interestingly, an increase in diversity was observed in HA rats.

A PCoA ordination of a Euclidean distances' matrix was used to visualize the microbial community structure in both groups (β-diversity) (Figure 1D). Clear cluster separation, primarily driven by PCoA1, was evident between the control and HA rats (PERMANOVA, *R*^2^= 0.27840, *p* < 0.001).

### 3.2 HA alters gut microbial taxa and increases microbiome network complexity in rats

**Figure 2:**
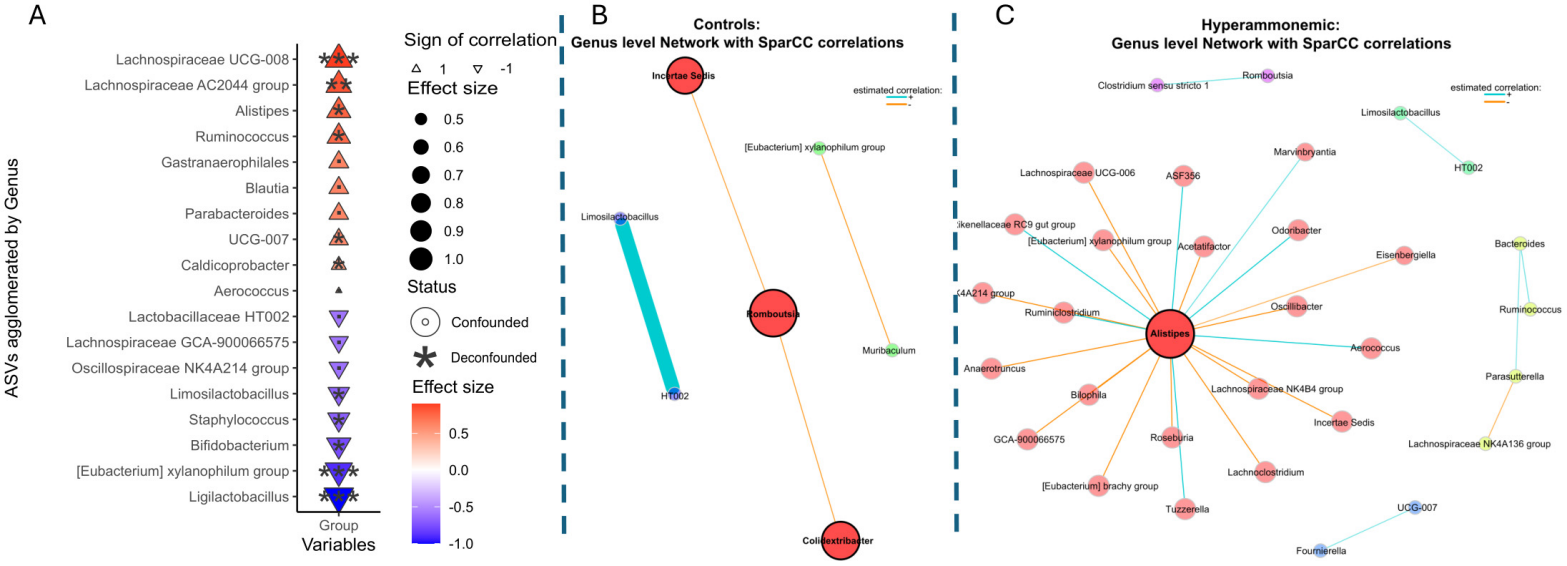
Relevant genera in the hyperammonemic group. **(A)** Genus associations with the HA rats. Color scale and size illustrate the effect size and the sign of correlation is represented by the direction of the triangular shape (up = positive, down = negative). Significance is denoted by black asterisks based on FDR-adjusted *p*-values (FDR-values: <0.001 = ***, <0.01 = **, <0.05 = *, <0.1 =.). **(B)** Network map showing significant relationships between genera in the control group and in the **(C)** HA rats' group. Edges shown between nodes are those with Benjamini–Hochberg false discovery rate <0.05. Color scale shows positive (blue) or negative (orange) significant correlations. Nodes with a higher degree centrality are larger.

The genera *Alistipes* and *Ruminococcus*, as well as some species belonging to the *Lachnospiraceae* family, were enriched in the faecal samples from HA rats. In contrast, the abundance of *Eubacterium xylanophilum* (group) and lactic acid bacteria belonging to the *Ligilactobacillus, Limosilactobacillus* and *Bifidobacterium* genera decreased significantly (Figure 2A). When descending to the species level, ASVs belonging to *Lachnospiraceae* (UCG008), *Alistipes putredinis* and *Bacteroides cellulosilyticus* were predominant in the HA GM, and ASVs with a reduced presence corresponded mainly to the species *Ligilactobacillus murinus*, and to a lesser extent other Lactobacillaceae such as *Limosilactobacillus reuteri*, as well as *Bifidobacteribacterium pseudolongum* or *Staphylococcus xylosus* (Supplementary Figure 1). Further tests using ANCOM-BC supported these results (Supplementary Figure 2).

Network analysis outlines significant positive and negative microbial correlations. Here, it was used to examine the different interactions between bacterial genera in control and HA rats, and to identify the genera positioned at the main nodes of interaction. Networks for control and hyperammonemic groups contained 7 and 32 significant nodes (genera), and 4 and 27 edges (links that connect nodes), respectively, representing significant microbial associations. Control rats had fewer significant interactions at the genus level, with 3 keystone genera (*Romboutsia, Colidextribacter* and an unidentified species in the family Ruminococcaceae) which were positively interrelated (Figure 2B). In HA rats, *Alistipes* genus showed the highest centrality, with complex associations (Figure 2C). Both groups exhibited a positive interaction between *Limosilactobacillus* and the HT002 genus from the *Lactobacillaceae* family, indicating a common interaction in rats.

Network robustness analyses showed that, across 200 bootstrap resamplings, the HA network consistently showed higher density than Controls (median (Q1, Q3): 0.0053 (0.0003, 0.012) vs. 0.0009 (0.0003, 0.0226); Wilcoxon p = 8.98 × 10^−9^). Although permutation-based null model testing (*n* = 500; 336 successful) comparing single-network densities did not reach significance due to high variability in small networks, the bootstrap results indicate that increased density in HA is a, supporting the robustness of this observation.

### 3.3 Microbial NH_4_-asociated metabolic pathways are affected in the gut of HA rats

**Figure 3:**
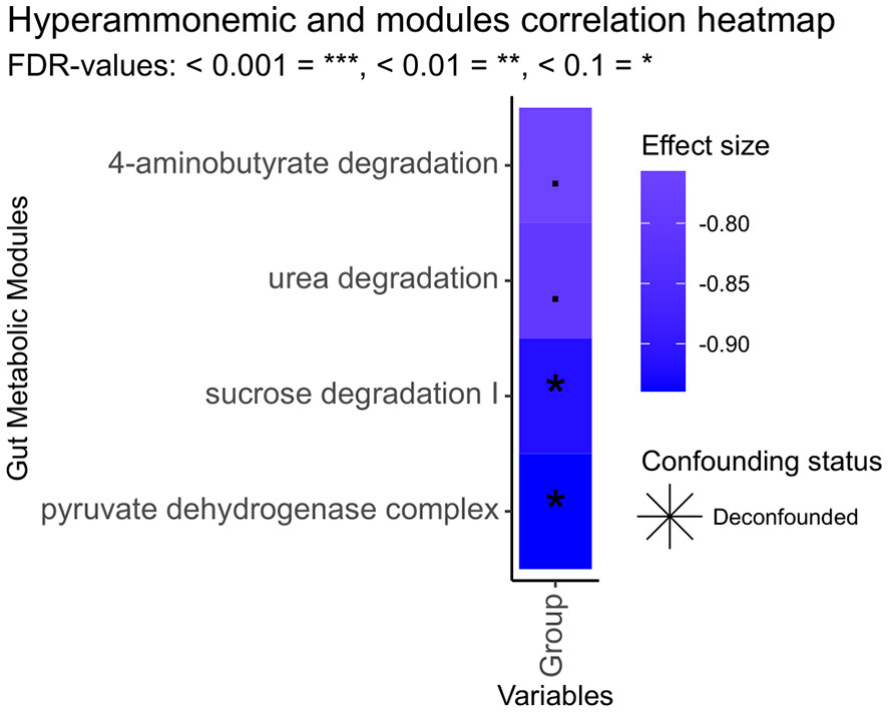
GMMs associations with hyperammonemic group. Color scale and size illustrate the effect size (dark blue = −1, red = 1). Significance is denoted by black asterisks based on FDR-adjusted *p*-values: <0.1 =., <0.05 = *.

**Figure 4:**
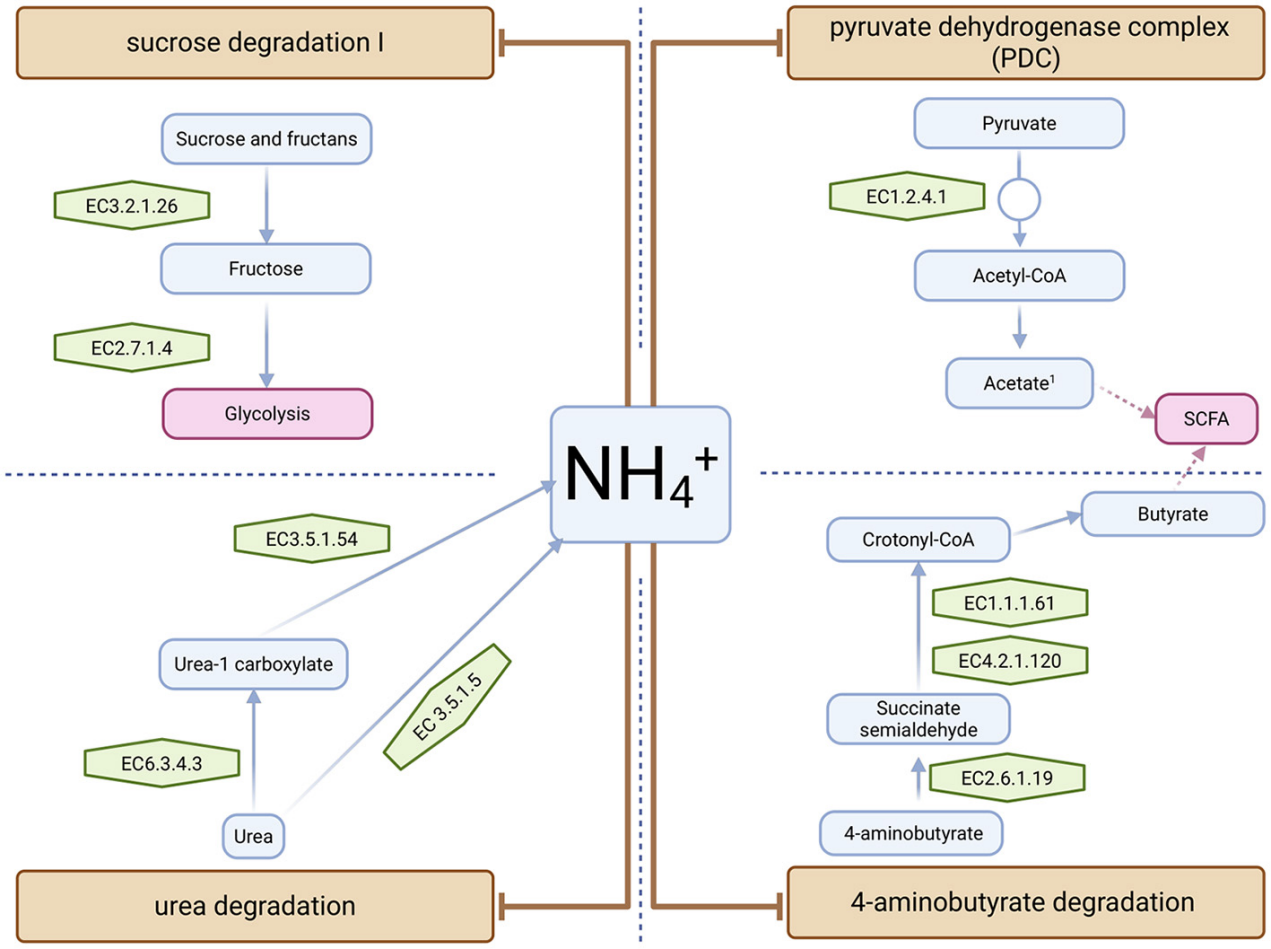
Scheme showing the probable effect of ammonium (NH_4_^+^) depressing specific gut metabolic modules (GMMs) in hyperammonemic rats. Brown boxes show GMMs, blue boxes denote metabolites, green boxes represent enzymes associated with the GMMs and light red boxes describe affected pathways. Brown arrows indicate the inhibitory effect of elevated NH_4_^+^ levels in the gut on GMMs and derived bacterial pathways. Inferred enzymes from the Kegg Orthology codes (parenthesis) inferred with PICRUSt2 and Omixer-RPM algorithms: EC 3.2.1.26, beta-fructofuranosidase (K01193); EC 2.7.1.4, fructokinase (K00847); EC 1.2.4.1, pyruvate dehydrogenase complex (K00161, K00162, K00163, K00382, K00627); EC 2.6.1.19, 4-aminobutyrate aminotransferase (K00823, K07250, K13524, K14268); EC 4.2.1.120, 4-hydroxybutyryl-CoA dehydratase (K14534); EC 1.1.1.61, 4-hydroxybutyrate dehydrogenase (K08318); EC6.3.4.6, urea carboxylase (K01941); EC 3.5.1.54, allophanate hydrolase (K01457); EC 3.5.1.5, urease alpha, beta and gamma subunits (K01427, K01428, K01429, K01430, K14048). Created using BioRender.

The metabolic activity of the gut microbiota can be inferred from available genomes using the PICRUSt2 algorithm. This bioinformatics tool paired to omixer-RPM allows the identification of the predominant functional units of the GM, also called gut metabolic modules (GMMs), which are gene sets that encode enzymes connected to metabolic pathways. When the metabolic potential of the microbiome in fecal samples from HA rats was compared to that of control rats, a negative association was found between HA and specific GMMs, including urea and sucrose degradation, the pyruvate dehydrogenase complex and 4-aminobutyrate, also γ- aminobutyrate (GABA), degradation (Figure 3). A detailed study of these GMMs and the gene orthology in the Kyoto Encyclopedia of Genes and Genomes (KEGG, https://www.genome.jp/) revealed detailed microbial metabolic pathways and enzymes that are significantly affected by HA (Figure 4).

The *pyruvate dehydrogenase complex* includes pyruvate dehydrogenase and, under anaerobic conditions, possibly pyruvate-formate lyase bacterial activities (EC1.2.4.1). These pathways generate acetyl-CoA, which is required for the formation of acetate (Figure 4). These activities are typically found in fermentative bacteria such as lactic acid bacteria and bifidobacteria, which were negatively correlated with HA (Figure 2). Under anaerobic conditions, specialized gut microbiota can transform acetyl-CoA into acetate and, via acetoacetyl-CoA, to SCFA, such as butyrate (Louis and Flint, 2017). Consequently, HA could interfere with SCFA production.

The GMM *sucrose degradation I* was also identified as being negatively correlated with HA (Figure 3). Searching for associated orthologous genes revealed that the levels of invertase-like beta-fructofuranosidase (EC3.2.1.26) and fructokinase (EC2.7.1.4) enzymes were significantly lower in the presence of NH4^+^ (HA). These results suggest impaired fructan utilization, including sucrose and potentially oligofructosaccharides, as well as decreased glycolytic capacity.

Ammonium is a product of bacterial urease (EC 3.5.1.5), urea carboxylase (EC6.3.4.6) and allophanate hydrolase (EC:3.5.1.54) activities. As consequence, these activities are repressed in the presence of ${\text{NH}}_{4}^{+}$, which will directly affect the urea cycle and adjacent pathways. Inhibition of the *urea degradation* (MF0085) in gut bacteria affects different metabolic pathways through the accumulation of ${\text{NH}}_{4}^{+}$, which increases the formation of carbamoyl phosphate. Carbamoyl phosphate is used for arginine biosynthesis, degradation of xenobiotics such as the herbicide atrazine, and purine metabolism and its secondary metabolites.

Finally, the *4-aminobutyrate degradation* GMM was also significantly reduced in HA rats. This module included the following enzymatic activities: 4-aminobutyrate aminotransferase (EC2.6.1.19), 4-hydroxybutyryl-CoA dehydratase (EC:4.2.1.120) and 4-hydroxybutyrate dehydrogenase (EC1.1.1.61). These enzymes are typically found in *Firmicutes* which produce crotonyl-CoA, a precursor of butyrate.

### 3.4 Metabolite analysis supports the differences in GMM

**Figure 5:**
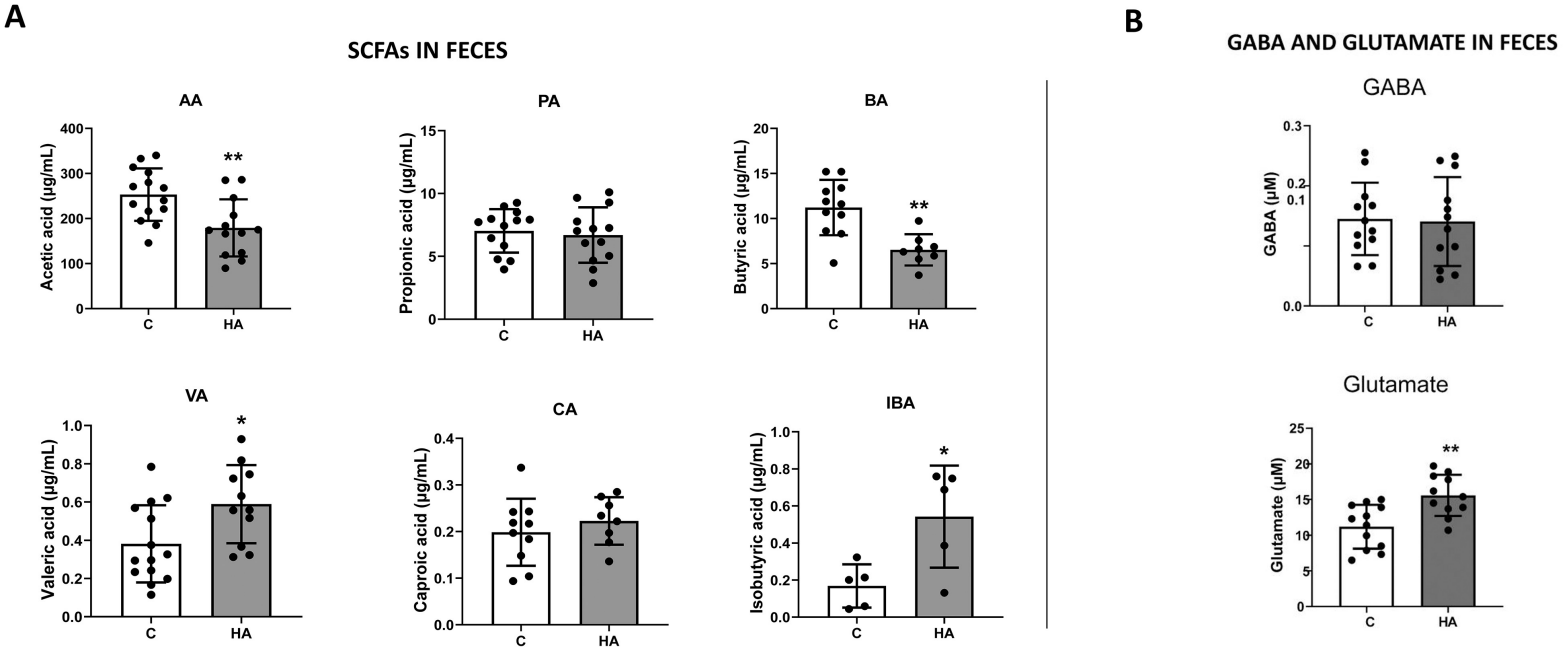
Fecal metabolites of HA vs control rats. These graphs show mean±SD of the concentrations of **(A)** SCFAs, including acetic (AA), propionic (PA), butyric (BA), valeric (VA), caproic (CA) and isobutyric (IBA) acids and of **(B)** 4-aminobutyrate (GABA) and glutamate in fecal samples of HA and control rats. (*) *p* < 0.05; (**) *p* < 0.01.

SCFA concentrations were determined in the fecal samples of the rats. As predicted, the HA group had significantly lower levels of acetic and butyric acid, confirming the inhibition of acetate formation from the *pyruvate dehydrogenase complex* and butyrate synthesis from both acetate/acetyl-CoA and the degradation of *4-aminobutyrate* (GABA) (Figure 5A); however, no differences were found in GABA concentrations between controls and HA rats (Figure 5B). In addition, the hyperammonemic group showed higher concentrations of valeric acid (pentanoic acid) and isobutyric acid (2-methylpropanoic acid) (Figure 5A). Both require an initial source of pyruvate; however, valeric acid is synthesized from 2-ketobutyrate and requires the activity of leucine synthesis enzymes (LeuABCD) (Dhande et al., 2012), while isobutyric acid is synthesized by bacteria from acetolactate using valine synthesis enzymes (IlvCD) and the Ehrlich pathway (Li et al., 2011). As mentioned above, there was no difference in GABA concentrations, but HA rats showed a significant increase of fecal glutamate (Figure 5B). This higher concentration of glutamate may be correlated with the decrease in gut lactobacilli that produce an enzyme called glutamate decarboxylase that catalyzes the decarboxylation of glutamate to GABA (Yogeswara et al., 2020).

In an independent analysis, and with a different batch of rats, amino acids concentrations, such as glutamine, serine, arginine and citrulline, all related to nitrogen metabolism or gut microbiota metabolism, were determined in plasma of HA and control rats. HA rats had significantly higher concentrations of glutamine and arginine and lower serine in serum possibly due to diffusion of gut metabolites (Supplementary Figure 3). Tryptophan and its metabolites, a pathway also modulated by gut microbiota, were also measured in plasma, where HA rats had lower concentrations of tryptophan, kynurenine, serotonin and xanthurenic acid (Supplementary Figure 4).

## 4 Discussion

Chronic HA is a major contributor to cognitive and motor impairments observed in cirrhotic patients with HE. In cirrhosis, HA arises from impaired urea cycle function in the injured liver, while ammonia itself is generated through amino acid degradation in the small intestine and by colonic bacteria that degrade amino acids or express urease. In this context, our study aimed to investigate whether increased intestinal ammonia contributes to alterations in the gut microbiota (GM) and its metabolic functions. Our findings demonstrate that HA induces significant changes in GM composition and function, which may underlie the peripheral and neuroinflammatory processes associated with HE.

Previous studies have shown heterogeneous results regarding microbiome diversity in the context of liver disease. For example, α-diversity increased in patients with cirrhosis and hepatitis B virus (HBV)-related liver disease (Zheng et al., 2020; Lin et al., 2023), while a decrease was also observed in patients with liver disease and in rat models (Bajaj et al., 2020; Yang et al., 2023). Furthermore, no significant differences in α-diversity were found in other cases of HBV–induced chronic liver disease (Zeng et al., 2020). In our study, we found no significant differences regarding the Shannon index.

Network analysis highlighted the central role of the genus *Alistipes* in HA. This genus has recently been associated with cancer and various non-communicable diseases, most notably with major depressive disorder (Parker et al., 2020). Additionally, *Lactobacillus* and certain *Bifidobacterium* species produce GABA from glutamate (Barrett et al., 2012; Yogeswara et al., 2020). Interestingly, fermentative bacteria, such as lactobacilli (*Ligilactobacillus* and *Limosilactobacillus*), *staphylococci* and bifidobacteria are decreased in HA rats.

GMM analysis of HA *vs*. control rats revealed that ammonia strongly influences gut microbiome metabolic functions, which were inferred from genomic databases after 16S rRNA gene sequencing. There is a strong negative correlation between HA and the *pyruvate dehydrogenase complex*. This bacterial module is associated with the reduction of lactic acid and fermentative bacteria because this enzyme complex is a known major crossroads pathway in the lower glycolysis in this bacterial group. A direct consequence of the reduction in the activity of *pyruvate dehydrogenase complex* is the reduced production of acetyl-CoA, acetic acid from carbohydrate fermentations. These compounds are substrates for butyrate-producing commensal bacteria (Louis and Flint, 2017). Butyrate produced by the gut microbiome provides energy to the epithelial cells and restores gut permeability (Singh et al., 2023). Also in animal models, butyrate reduces neuroinflammation induced by quinolinic acid (Ge et al., 2023), improves vascular dementia (Liu et al., 2015) and Alzheimer's disease (Wang et al., 2022). The reduction of butyrate in stools observed in HA rats is directly related to the symptoms described of this rat model (Balzano et al., 2020).

In HA rats, the negative correlation of *4-aminobutyrate*-or γ*-aminobutyrate- (GABA) degradation pathway* is complementary to the increase of GABA biosynthesis. Increased extracellular GABA in the cerebellum of HA rats induces motor incoordination and GABA signaling alteration in cerebellum or hippocampus are also involved in cognitive dysfunction in HA rats (Cauli et al., 2009b; Sancho-Alonso et al., 2022). Altered GABA signaling in the cerebellum of HA rats is caused in part by cerebellar neuroinflammation (Cabrera-Pastor et al., 2018; Balzano et al., 2020). The influence of GM on the GABA levels of the brain was demonstrated in germ-free mice which were colonized with GM of cirrhotic patients. The colonized mice exhibited increased GABA levels in the cerebral cortex compared to those colonized with stool from healthy donors (Liu et al., 2020). GABA stimulates the vagus nerve, which affects brain function (Bravo et al., 2011). Our results suggest that the increase in GABA levels in HA can be attributed to the growth of *Alistipes* and *Bacteroides* populations, as previously mentioned. However, the abundance of *Lachnospiraceae* in HA differs from findings in prior studies on the relationship between gut microbiota and depression (high GABA levels) (Naseribafrouei et al., 2014). Therefore, changes in GM in HA rats may increase GABA levels, activating GABA receptors in immune cells and contributing to peripheral inflammation and, eventually, neuroinflammation (Balzano et al., 2020). Furthermore, reduction of this predicted GMM could also decrease the synthesis of butyrate due to the reduced formation of succinate semialdehyde and crotonyl-CoA (Figure 4). As mentioned above, butyric acid is very relevant metabolite required for brain function, intestinal integrity and general health (Balzano et al., 2020).

Urea synthesis in the liver detoxifies ammonia excess, in fact in HA rats, urea levels are also increased due to increased ammonia as these rats have not compromised liver function and urea cycle (Felipo et al., 1988). Our results suggest that the reduced *urea degradation* by the GM of HA rats may also contribute to the previously observed increase of urea levels. The enzyme urease, that converts urea to ammonia and carbamate, is a common bacterial enzyme often associated to virulence. Urease is repressed by nitrogen sources like ammonium (Collins and D'Orazio, 1993), which fully agrees with the finding of a likely decrease in the GMM *urea degradation* in HA rats. In the lactic acid bacterium *Streptococcus thermophilus*, urease activity strongly induces glycolysis as ${\text{NH}}_{4}^{+}$ is a powerful inducer of phosphofructokinase (Arioli et al., 2022). In an environment where mono and disaccharides are limited, such as the rat intestine, boosting the activity of glycolytic enzymes could quickly exhaust fermentable substrates.

The reason why specific bacterial groups are inhibited in the gut of HA rats can be related to specific reactions inhibited by ammonia. Excess ${\text{NH}}_{4}^{+}$ may kidnap pyruvate to form serine, thereby deactivating pyruvate dehydrogenase and various deaminases, such as methyl aspartase and glutamate dehydrogenase (Buckel, 2001). Carbamate kinase catalyzes the reaction (ATP + NH_3_ + CO_2_ ↔ ADP + carbamoyl phosphate), and it is a widely distributed enzyme among bacteria (Minotto et al., 2000). Carbamate kinase is part of the arginine and polyamine deiminase pathways. ${\text{NH}}_{4}^{+}$ can reverse this reaction, resulting in ATP depletion in bacterial species with efficient carbamate kinases, such as lactobacilli and bifidobacteria. This may explain that their proliferation is restricted in the intestine of HA rats (Cunin et al., 1986; Zúñiga et al., 2002; Nakada and Itoh, 2003). In addition, the inferred *sucrose degradation I* module comprises early steps in mono and disaccharide assimilation pathways, which are essential in these fermentative bacteria. They lack respiratory functions and utilize glycolytic products as final electron acceptors, thereby accumulating tricarboxylic acids, such as lactate and acetate (Pérez-Martínez et al., 2008).

Carbamoyl phosphate kinase (CPK) and carbamoyl phosphate synthase (CPS) are key bacterial enzymes in converting ammonia to carbamate, which requires ATP. The resulting carbamate is then used by transaminases for amino acid synthesis (He et al., 2023). In the arginine deiminase pathway (ADI), CPK reaction is reversible generating ${\text{NH}}_{4}^{+}$ and ATP from carbamoyl phosphate (Zúñiga et al., 1998). Arginine and aminated amino acids, such as glutamine, asparagine, tryptophan and histidine, provide the main source of nitrogen for protein synthesis (Wu et al., 1999; Schimmel et al., 2023). Ammonia accumulation will directly stimulate the formation of carbamoyl phosphate, which serve as a source of amine groups in amino acid synthesis (He et al., 2023), while also blocking amino acid transport and metabolism (Simen et al., 2017). As consequence, excess of ammonia stimulates the synthesis of carbamoyl phosphate by CPK and CPS, which can deplete bacterial cell's ATP. The effects of ammonia on bacterial predicted pathways explain its observed inhibitory effect on the growth of certain bacterial species (Sissons et al., 1990).

Other consequences of excess ammonia are related to the metabolism of glutamate and synthesis of GABA which impact host health. Amino acid metabolism and ammonia assimilation are connected to GABA synthesis via glutamate. Glutamine synthase fixes ammonium to render glutamine (Schimmel et al., 2023), while glutamate decarboxylase produces GABA. The synthesis and secretion of GABA via the glutamate/GABA antiporter is stimulated by acid stress and this process occurs in all bacterial species studied (Feehily and Karatzas, 2013). However, no difference was found in the concentrations of GABA detected in the feces of HA rats, though a significant increase in glutamate was observed. Interestingly, hyperammonemia in rats has been associated with higher levels of extracellular glutamate in the brain and altered neurotransmission (Cabrera-Pastor et al., 2019). Furthermore, trophic amino acids, such as threonine, arginine, and glutamine, are also essential for the health of intestinal epithelial cells (Bortoluzzi et al., 2018).

Non-alcoholic fatty liver disease (NAFLD), now called metabolic dysfunction-associated steatotic liver disease (MASLD), is the most common disease related to sucrose intake (Sigma, 2022). Furthermore, a high-sucrose diet in mice and rats down-regulates hepatic endoplasmic reticulum stress adaptive pathways and leads to lipid accumulation and hepatic steatosis (Flister et al., 2018; Sousa et al., 2018). Our results suggest that reduction of sucrose degradation in the GM of HA rats could favor higher sucrose availability in the cecum.

Finally, the *Alistipes* genus was the central node/keystone genus in HA rats. This finding is consistent with previous studies describing *Alistipes* as playing a potential pathogenic role in diseases such as anxiety, myalgic encephalomyelitis/chronic fatigue syndrome, depression, autism and colorectal cancer. The *Alistipes* genus could play a leading role in disease modulation or act as a co-inducer of diverse clinical phenotypes (Parker et al., 2020).

Some limitations were encountered in this study. First, GMMs were generated through inference from the 16S rRNA gene sequencing data; to support the results obtained, levels of SCFAs, GABA and glutamate were determined in feces. Only male rats were used as this model is already well established in male animals. Behavior tests, food intake and body weight after treatment were not recorded because this model has already been widely described and used (Azorín et al., 1989; Johansson et al., 2015; Cabrera-Pastor et al., 2016; Hernández-Rabaza et al., 2016; Taoro-González et al., 2019). Changes in neurotransmitters, such as GABA and glutamate, vary in different cerebral areas of hyperammonemic rats as already reported (Cauli et al., 2009a; Cabrera-Pastor et al., 2018; Sancho-Alonso et al., 2025); hence, here only neurotransmitters in feces were analyzed.

With the presented consistent results (inferred GMM and fecal SCFA quantification), we propose that elevated intestinal ammonia levels resulting from hyperammonemic diet affect the downregulation of GM pathways such as GABA/glutamate metabolism and butanoate, TCA cycle, urea degradation and sucrose degradation. All of them can contribute to HE. Our results are consistent with the functional and taxonomic changes observed in previous studies. This concordance highlights the robustness of our HA rat model, to study contribution of HA to changes in the GM which are associated to alterations leading to HE, and despite the differences between the human and rodent microbiomes, our model proves to be a reliable model for studying the microbiome in the context of liver disease.

## Data Availability

The datasets generated for this study can be found in the NCBI Sequence Read Archive (SRA), under accession number PRJNA1378829 (https://www.ncbi.nlm.nih.gov/bioproject/PRJNA1378829). All other data are included in the study in the Supplementary Data section. The scripts used for the analysis can be found at https://github.com/lolapsgp/Rats_HE_HiperAmmon_model.
