# Supplementary material for: Hyperammonemia reduces the populations of beneficial lactobacilli and bifidobacteria, disrupting the metabolic balance of the gut microbiome in rats
Source: Front Microbiol. 2026 Mar 27;17:1771709. doi: 10.3389/fmicb.2026.1771709 (PMC13067365; doi:10.3389/fmicb.2026.1771709)
Supplement: Supplementary file 1 [file Data_Sheet_1.docx]

Supplementary Material

Hyperammonemia reduces the populations of beneficial lactobacilli and bifidobacterial, disrupting the metabolic balance of the gut microbiome in rats

# Supplementary Data

**Supplementary Methods for Supp figures 2, 3 and 4**

ANCOM-BC was applied for differential abundance testing, at genus level and ASV level. BH method was used to adjust for p-values. Threshold for filtering samples based on library sized was set to 1000. Formula included both variables Group and Batch. It was indicated to detect structural zeros and to classify a taxon as a structural zero in the corresponding study group using its asymptotic lower bound. All other parameters were set as default.

Plasma metabolite concentration was analyzed as in Mincheva et al (2024).

The sample preparation for analysis of tryptophan (Trp) metabolites includes protein precipitation performed in 50 μL of plasma samples by addition of 10 μL of trifluoroacetic acid (SIGMA), mixture in vortex and centrifugation at 20.000 g for 15 min at 4 ºC. The supernatants were separated and 40 μL injected in the HPLC-MS. Luna Omega Polar C18 (OOD- 4760-AN) 100*2.1mm3 μm (100 A) column from Phenomenex, at 40 ºC, was used. The mobile phase consists of a two-phase gradient: 0.1% formic acid in water (A) and 0.1% formic acid in acetonitrile (B), as follows: 5% B 0–0.5 min, 5–80% B 0.5–5.0 min, 80% B 5.0–6.0 min, 80-5% B 6.0–6.1 min, 5% B 6.1–8 min, with a flow rate of 0.4 mL/min. ESI ion source in positive ionization mode was used with curtain gas 30, GAS1 40 and GAS2 60, 500 ºC and 4500 V in multiple reaction monitoring (MRM) mode with the following conditions for each metabolite: 1) Trp, 205.2 m/z > 146 , RT 2.5 EP 10, CE 30, DP 130, CXP 16; 2) kynurenine, 209.2 m/z > 192 and 94 m/z, RT 2.5 EP 8 CE 15 DP 90 CXP 15; 3) kynurenic acid, 190.1 m/z > 144 and 116 m/z, RT 2.5 EP 10 CE 25 DP 50 CXP 10; 4) xanthurenic acid, 206.2 m/z > 160 and 132 m/z RT 2.5 EP 10 CE 28 DP 130 CXP 16 and 5) serotonin (5-HT), 177.1 > 160 RT 1.7 EP 10 CE 12 DP 80 CXP 13. A standard curve from 500 to 300000 nM of Trp, from 5 to 3000 nM of kynurenine, from 0.5 to 300 nM of kynurenic and xanthurenic acids and from 50 to 30000 nM of 5-HT was prepared in H2O and processed as the samples to calculate metabolite concentration in samples.

For the analysis of the ammino acid the sample preparation was the same as for Trp metabolites, using the same chromatographic column. The mobile phase consists of a two-phase gradient: 0.1% formic acid in water (A) and 0.1% formic acid in acetonitrile (B), as follows: 20% B 0–0.5 min, 20–80% B 0.5–5.0 min, 80% B 5.0–6.0 min, 80-20% B 6.0–6.1 min, 20% B 6.1–8 min, with a flow rate of 0.4 mL/min.. ESI ion source in positive ionization mode was used with curtain gas 30, GAS1 50 and GAS2 55, 550 ºC, 4500 V and entrance potential of 10, MRM mode. Retention time was 0.6 for all metabolites with the following conditions for each ones: 1) Gln, 147>129.9, DP31, CE 15, CXP 8; 2) Glu, 148 > 84 DP 41, CE21, CXP 6; 3) Ser, 106 > 60 and 42, DP40, CE 20, CXP 4; 4) Arg, 175 > 60 and 130, DP 40, CE 30, CXP 8 and 5) Citrulline, 176 > 70, DP 40, CE 18, CXP 8. A standard curve from 0.01 to 100 µM of all the amino acids was prepared in H2O and processed as the samples to calculate its concentration in samples.

*Mincheva G, Felipo V, Moreno-ManzanoV, Benitez-Páez A, Llansola M. Extracellular vesicles from mesenchymal stem cells alter gut microbiota and improve neuroinflammation and motor impairment in rats with mild liver damage. Neurotherapeutics 2024. e00445. doi: 10.1016/j.neurot.2024.e00445*

# Supplementary Figures and Tables

##
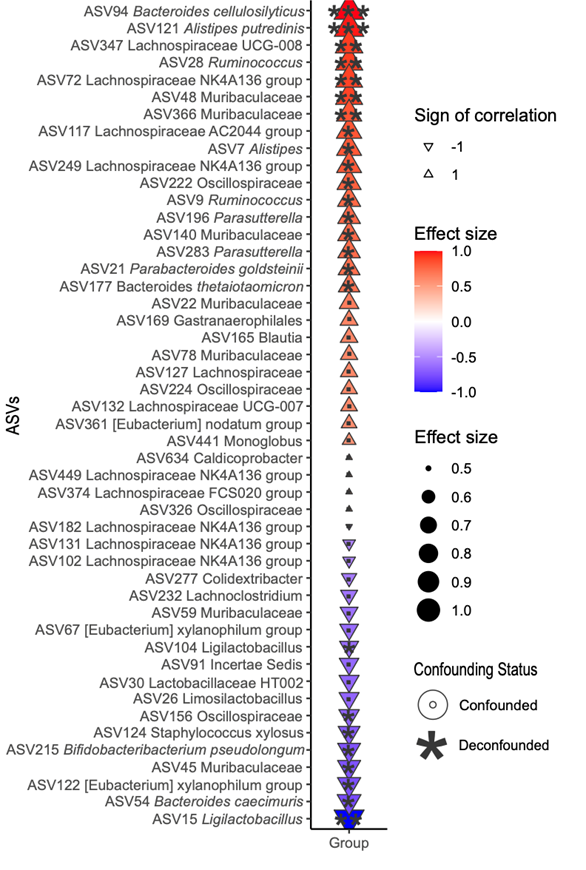
Supplementary Figures

## Supplementary Figure 1. ASV associations with the HA rats. Color scale and size illustrate the effect size and the sign of correlation is represented by the direction of the triangular shape. Significance is denoted by black asterisks based on FDR-adjusted p-values (FDR-values: < 0.001 = ***, < 0.01 = **, < 0.1 = *).


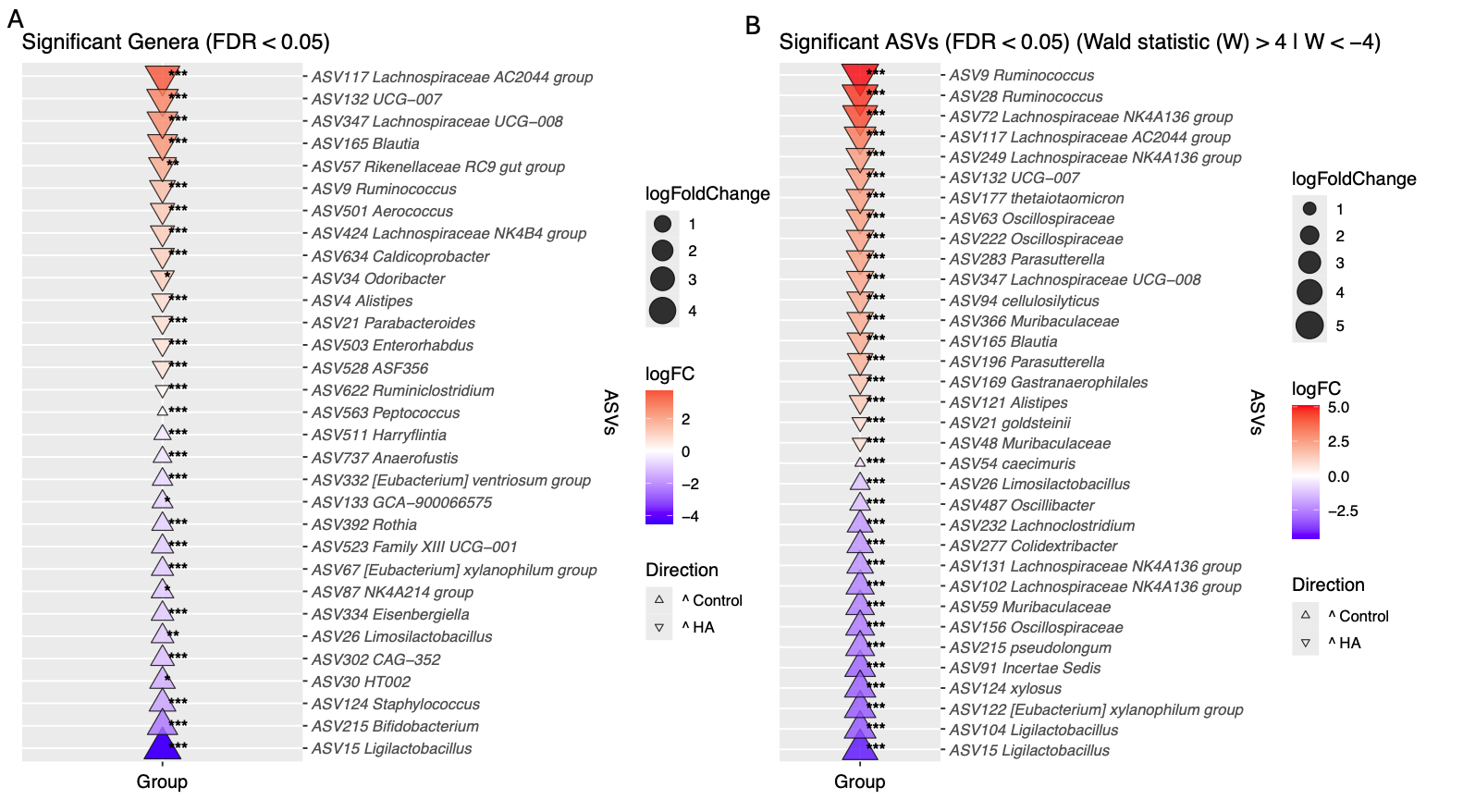


**Supplementary Figure 2.** Genera (A) and ASV (B) differentially abundant when comparing Control rats with HA rats using ANCOM-BC. Color scale and size illustrate the log Fold Change and the sign of correlation is represented by the direction of the triangular shape (Up higher in control; Down higher in HA rats). Significance is denoted by black asterisks based on BH-adjusted p-values (P-values: < 0.001 = ***, < 0.01 = **, < 0.1 = *).


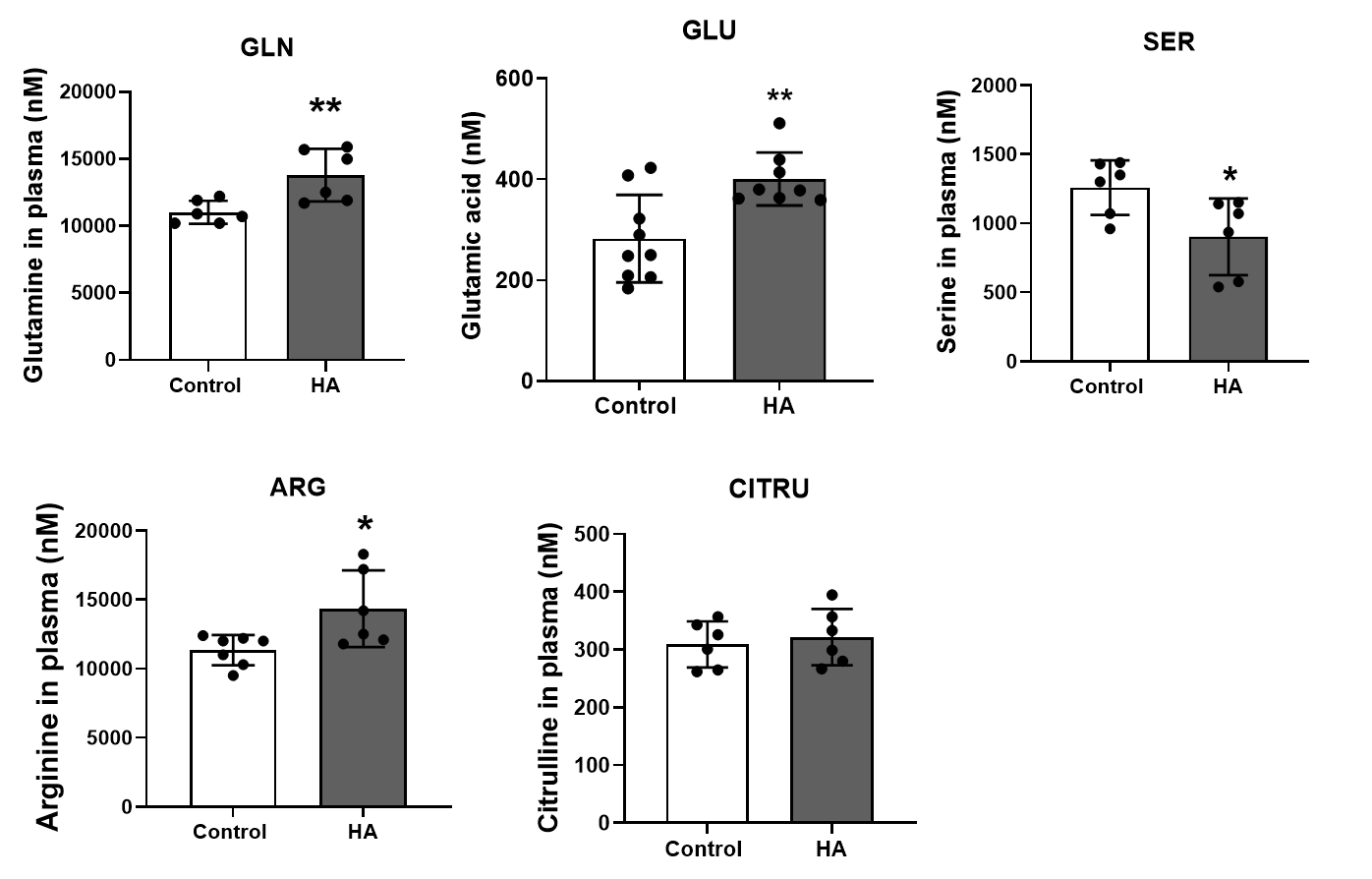
**Supplementary Figure 3.** Plasma amino acids in HA vs control rats. These graphs show mean±SD of the plasma concentration of glutamine (GLN), arginine (ARG), citrulline (CITRU), serine (SER) in plasma samples of HA and control rats. (*) p<0.05; (**) p<0.01.


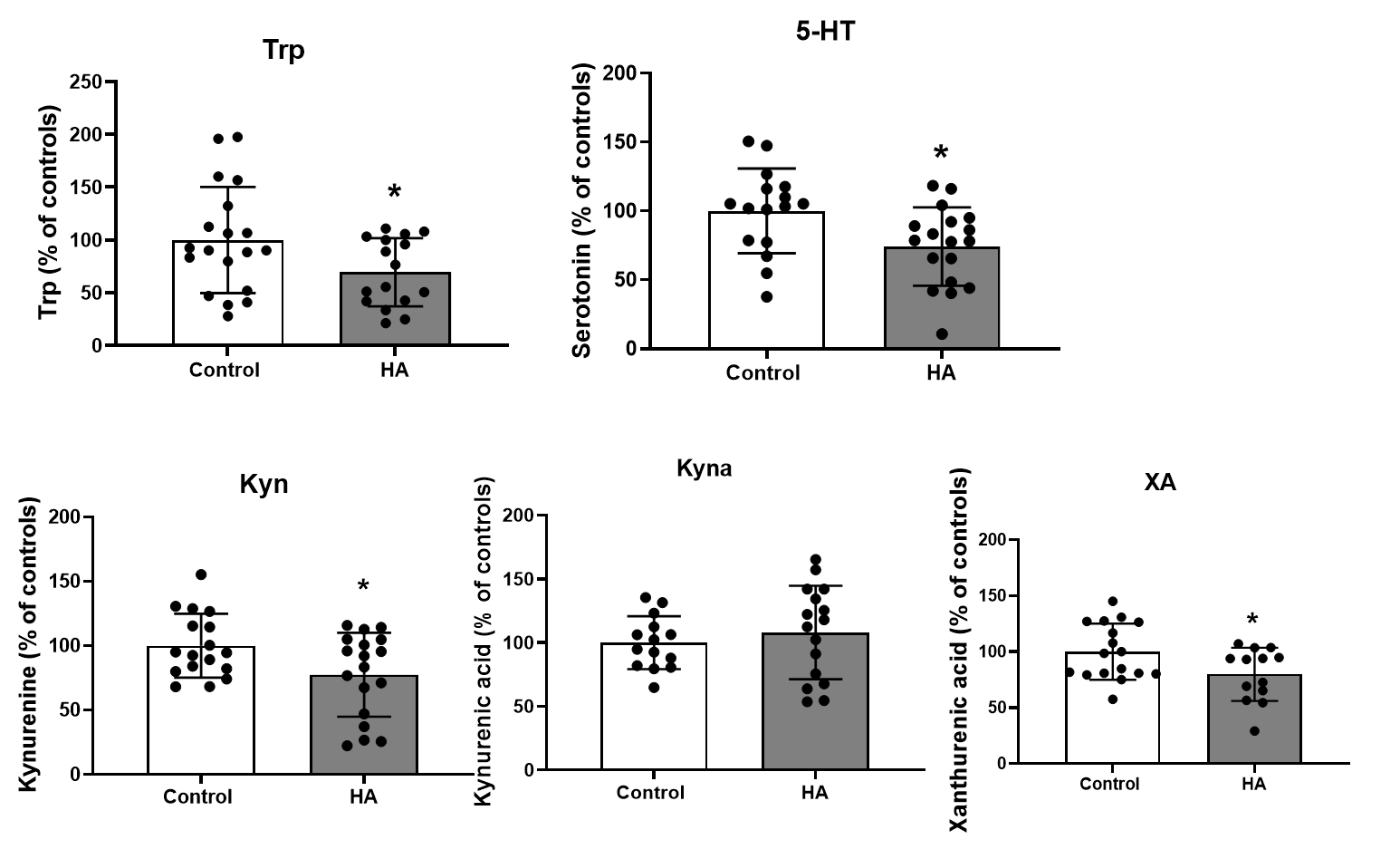


**Supplementary Figure 4.** Tryptophan metabolism metabolites in plasma from control and HA rats. Values represent metabolite levels (mean±SD), expressed as percentage of the control group. (*) p<0.05.
